# Effectiveness and safety of generic version of abacavir/lamivudine and efavirenz in treatment naïve HIV-infected patients: a nonrandomized, open-label, phase IV study in Cali-Colombia, 2011–2012

**DOI:** 10.1186/s12879-016-1871-x

**Published:** 2016-10-03

**Authors:** Jaime Galindo, Pedro Amariles, Héctor F. Mueses-Marín, Jaime A. Hincapié, Sebastián González-Avendaño, Ximena Galindo-Orrego

**Affiliations:** 1 Grupo Educación y Salud en VIH/SIDA, Corporación de Lucha Contra el Sida, Santiago de Cali, Colombia; 2 Grupo Promoción y Prevención Farmacéutica, Universidad de Antioquia (UdeA), Calle 70 No 52-21, Medellín, Colombia; 3 Grupo de Investigación en Atención Farmacéutica de la Universidad de Granada, Granada, España

**Keywords:** HIV/AIDS, Generic drugs, Antiretroviral drugs, Phase IV study

## Abstract

**Background:**

Generic drug policies are often associated with concerns about the quality and effectiveness of these products. Phase IV clinical trials may be a suitable design to assess the effectiveness and safety of generic drugs. The objective of this study was to describe the effectiveness and the safety of the generic abacavir/lamivudine and efavirenz in treatment-naïve HIV-infected patients.

**Methods:**

A monocentric, nonrandomized, open-label, phase IV study in treatment naïve HIV-infected patients 18 years or older with indication to receive abacavir/lamivudine and efavirenz were recruited from a program that provides comprehensive outpatient consultation and continuing care. The primary end-point was to achieve viral load <40 copies/mL at 12 months after baseline to assess effectiveness. Secondary end-point of the study were 1) to asses increasing in T-CD4 lymphocytes levels as accompaniment to asses effectiveness, and 2) to assess both gastrointestinal, skin, and central nervous system symptoms, and lipid profile, cardiovascular risk, renal, and hepatic function as safety profile. Data were determined at baseline, 3, 6, and 12 months. Close clinical monitoring and pharmaceutical care were used for data collection. Wilcoxon matched-pairs signed-rank test was used to compare proportions or medians.

**Results:**

Sixty patients were invited to participate in the study; 42 were enrolled and 33 completed the follow-up. Of the nine patients excluded from the study, only one was withdrawn due to adverse events. At 12 months, 31 of 42 patients (73.8 % in intention-to-treat analysis) achieved a viral load of HIV1 RNA <40 copies/mL. There was a significant increase (172 cells/mm^3^) in the median for CD4 T lymphocyte count. The adverse events were mild and met the safety profile for this antiretroviral regimen, mainly of central nervous system symptoms, skin rash, lipid abnormalities, and an increase of 2 % in the median of the percentage of cardiovascular risk.

**Conclusions:**

The clinical outcomes of generic version of abacavir/lamivudine and efavirenz in HIV treatment naïve patients showed the expected safety and effectiveness profile of proprietary ARV drugs.

**Trial registration:**

Registro Público Cubano de Ensayos Clínicos (RPCEC) ID: RPCEC00000202. Registered 19 November 2015.

## Background

Substantial progress has been made in the prevention and treatment of HIV/AIDS but it remains a public health challenge. A report on the global AIDS epidemic 2013 informed that in 2012 there were 35.3 million people living with HIV, 2.3 million new infections, and 1.6 million AIDS-related deaths [1]. It is well known that people with HIV/AIDS need comprehensive care, including the use of antiretroviral therapy (ART) which extends the lives of HIV patients and reduces the possibility of infecting other people [2]. Therefore, availability and access to safe and effective antiretroviral (ARV) drugs are important requirements for appropriate medical care for this group of patients [3].

The evidence-based clinical guidelines for treating HIV/AIDS infection in Colombia recommended the regimen abacavir/lamivudine (ABC/3TC) and efavirenz (EFV) as the preferred first line, which has been assed as a cost-effectiveness ARV treatment in the Colombia settings [4]. In addition, although in the World Health Organization (WHO) evidence-based clinical guidelines on the use of ARV drugs, the preferred first line regimen is tenofovir/ lamivudine (or emtricitabine) and EFV, the scheme ABC/3TC and EFV is an alternative for first-line regimen [5]. Both the Colombia [4] and WHO [5] guidelines stated that in patients with pre-treatment HIV RNA >100,000 copies/mL, the regimens ABC/3TC and EFV or ABC/3TC and atazanavir/ritonavir do not be used due to higher rates of virologic failure.

The development and consolidation of generic drug policy is a valid option for improving the availability of and access to ART [6]. On average, generic ARV drugs are sold at a cost roughly 30 % below that of original ARV drugs [7] and generics play an important role in price negotiation discussions with trademarked companies [8]. In addition, generic drug policy is recognized as a key strategy for price reduction ARV drugs a monopoly situation. In this way, some governments, for instance Brazil, had negotiated voluntary licenses with multinational pharmaceutical companies to promote generic competition and reduce prices. As consequence, in the case of atazanavir 150 mg, the cost per patient per year varied from US$ 2815.07 in 2005 to US$ 1150.35 in 2013 [9].

At the same time, generic drug policy is concerned with the quality and effectiveness of products, including ARV drugs [10]. Relate to quality, although bioavailability and bioequivalence studies are the typical to determine bioequivalence of generic drugs [11], dissolution testing and comparative studies in-vitro-in-vivo correlation are a mean to define a direct relationship between bioavailability of a drug and its in-vitro dissolution rate and are refereed as an option for this goal. Thus, this kind of studies are an important strategy to predict the in-vivo bioavailability and in some cases substituting clinical studies to determine bioequivalence [12]. In this way, in Colombia dissolution testing and comparative studies in-vitro-in-vivo correlation are considered as an alternative to clinical studies to determine bioequivalence for some generic drugs [13]. For instance, the drugs evaluated in this study, obtained the marketing authorization proved that there were not differences in dissolution behavior of dosage forms between innovators (reference products) and their generic counterparts (tested products), on March 2010 for ABC/3TC, and November 2006 for EFV. Thus, although bioequivalence of ABC/3TC and EFV generics have been determined according to Colombia regulations [13], these drugs did not have WHO prequalification medicines, which may add concerns about quality of these kind of products marketing in Colombia.

The results of effectiveness and safety in the clinical practice may contribute to generate trust in the quality of generic ARV drugs manufacturing in Colombia. Therefore, there is the need to obtain clinical results from HIV/AIDS patients who are on generic ART, under standardized conditions of medical practice [14]. Nonrandomized, open-label, phase IV studies can be designed and conducted to achieve this goal [14, 15]. In this type of study, close clinical monitoring and pharmaceutical care could be used to improve data collection and evaluation of the results regarding the safety and efficacy of ART [14, 16, 17]. Therefore, the objective of the present study was to describe the effectiveness and the safety of the generic of the generic version of ABC/3TC and EFV in treatment naïve HIV-infected patients attending a program that provides complete outpatient consultation and continuing care for patients with HIV/AIDS.

## Methods

### Research design

The present study was monocentric, nonrandomized, open-label, phase IV study in treatment-naïve HIV-infected patients 18 years or older with indication to receive combination antiretroviral therapy (cART) containing ABC/3TC and EFV.

### Patients and setting

Patients were recruited between January 2011 and November 2012 from Corporacion de Lucha Contra el Sida, Cali-Colombia, an institute that provides comprehensive outpatient consultation and continuing care of patients with HIV/AIDS. In Colombia, a previous study conducted in cohorts of patients with similar conditions showed a close to 20 % drop-out rate and a percentage of virologic failure close to 15 %, during first year on ART [14]. Thus, if we assumed a virologic failure rate of 15 %, a group of 33 patients are need to find at least five failures per treatment [18]. In addition, we estimated a withdrawal rate of 20 %, thus a group of 41 patients are need as a minimum sample size for the study.

### Inclusion criteria

Patient eligibility included: a) HIV diagnosed by laboratory testing (presumptive tests and confirmatory test, CD4 T-lymphocytes count, and viral load values); b) treatment naïve and no less than 18 years of age; c) met immunologic, virologic, or clinical criteria for starting cART, according to recommendations of the International AIDS Society–USA Panel of 2010 [19] regarding to abacavir/lamivudine and efavirenz scheme, such as: i) viral load (RNA copies) <100.000 copies/mL and regardless of CD4 cell count; ii) genotypic test negative for drug resistance, iii) cardiovascular (CV) risk assessed as low (<10 %), using the Framingham risk score [20] d) major histocompatibility complex allele (HLA-B*57) genotype negative; and e) absence of metabolic syndrome, diabetes, and current or past medical history of cardiovascular disease (CVD).

### Exclusion criteria

Patients with any of the following clinical condition were excluded: a) pregnant, lactating, or women of child-bearing age not using a contraception method; b) current or past medical history of renal failure (serum creatinine equivalent to an estimated creatinine clearance <60 mL/minute) or hepatic failure; c) serious anemia (Hb <7.0 g/dL); d) high-sensitivity C-reactive protein (hs-CRP) >2.0 mg/L and two of the following CV risk factors: 1) smoking, 2) total cholesterol (TC) ≥240 mg/dL, 3) triglycerides >200 mg/dL, 4) high-density lipoprotein cholesterol (HDL-C) ≤40 mg/dL, 5) glycemia > 110 mg/dL, or 6) large waist circumference and waist/hip ratio (men with waist circumference >102 cm and waist/hip ratio >0.9 or women with waist circumference >88 cm and waist/hip ratio >0.8); e) three of the 6 CV risk factors cited above; f) being treated with drug(s) with high probability of clinically relevant interactions (e.g., rifampicin, itraconazole); f) major psychiatric disorder; g) current or past medical history of substance abuse; or h) limitations to attending the visits.

All enrolled patients were assigned the generic cART regimen ABC/3TC 600/300 mg and EFV 600 mg taken once daily [19] (provided by Humax Pharmaceutical S.A., Medellin-Colombia) and were followed for 12 months.

The primary end-point was to achieve viral load <40 copies/mL at 12 months after baseline to assess effectiveness. Secondary end-point of the study were 1) to asses increasing in T-CD4 lymphocytes levels as accompaniment to asses effectiveness, and 2) to assess both gastrointestinal, skin, and central nervous system symptoms, and lipid profile, cardiovascular risk, renal, and hepatic function as safety profile.

### Effectiveness and safety assessments

Serum biochemistry: transaminases, creatinine, lipids, glucose, and hs-CRP, hematology (red and white blood cell differential count, platelet count, hemoglobin, and hematocrit), HIV-1 RNA viral load and CD4 lymphocytes count as medical history and physical examination, including measurements of waist and hip circumferences, were inclusion criteria for screening.

HIV-1 RNA load (RNA copies/mL) and CD4 cell count (cells/mm^3^) were determined at baseline, 3, 6, and 12 months, as clinical outcomes to assess effectiveness. In detail, effectiveness related to viral load was defined as a viral load <40 copies/mL measured at 12 months after baseline. Viral load was quantified by real-time reverse transcriptase PCR (RT-PCR) Abbott Real Time HIV-1 m2000 assay (Abbott, Chicago, IL). Blood samples were collected by a certified technician and processed in a centralized national reference laboratory. PCR testing was performed on frozen samples rather than as soon as blood was drawn.

To assess drug safety, liver tests (alanine aminotransferase and aspartate aminotransferase), renal tests (serum creatinine and estimated creatinine clearance, using the Cockcroft-Gault method), complete blood count (number of red blood cells, white blood cells, and platelet, hemoglobin, and hematocrit), and CV risk assessment, using the Framingham risk score [20], including glucose, hs-CRP, full lipid profile, and waist and hip circumferences were performed. Except for lipid profile and assessment of metabolic syndrome and CV risk, which were measured at baseline, and at months 6 and 12, all tests were performed at baseline, 3, 6, and 12 months. Patients were asked for adverse events at each visit. The most common adverse effects associated with ARV drugs (gastrointestinal, skin, and central nervous system [CNS] symptoms) were judged at each medical examination or appointment for pharmaceutical care.

### Monitoring and data collection

At each medical examination blood samples were taken for safety assessment; complete physical and clinical monitoring was performed by a physician trained and experienced in the care of persons with HIV/AIDS; and pharmaceutical care using the DADER method [21] was performed by a pharmacist trained in this practice. All data were recorded in the institution’s medical records.

The DADER method for pharmaceutical care is a systematic process developed by the Research Group of Pharmaceutical Care at the University of Granada, Spain. The intervention is based on the use of pharmacotherapy records, evaluation of an assessment form that includes all HIV/AIDS health problems and the drugs used to treat these medical problems, and their assessment on a specific date. Thus, pharmacists did the following [17, 21]:

- Obtained patient data related to HIV/AIDS medical problems and current drug therapy with special focus on adherence to treatment and detection of adverse events. Patient-specific data were collected by interviewing the patient and reviewing the drug and clinical records (mainly drug history and the results of clinical laboratory tests). During the first interview, 1 month’s supply of drugs for the cART regimen were dispensed and the patient received verbal counseling regarding cART, including the medication schedule and possible adverse effects. At weeks 1 and 2 and at the end of first month of cART follow-up calls were carried out. In addition, during the all-period of following patients may contact to pharmacist to ask any question related to some possible adverse event.
- Used the collected data to complete the assessment form, which was interpreted and evaluated once all the necessary information was added.
- Evaluated the patient’s drug therapy outcomes. The aim of this activity was to assess whether the desired treatment goals for HIV/AIDS were achieved. For patients whose goals were not yet achieved, the pharmacist developed therapeutic plans that included interventions with the aim of achieving the desired clinical outcome. In this way, pharmacist asked and for adverse events at each visit. So, both the most common adverse effects associated with ARV drugs (gastrointestinal, skin, and central CNS symptoms) and results of laboratory test were judged at each appointment for pharmaceutical care. If pharmacist identified some safety problem, he informed to clinician the safety and the physician valued and verified the safety problem.
- Conducted an intervention intended to directly prevent or resolve any potential or actual patient health outcomes that were not consistent with the objectives of pharmacotherapy and were associated with the use of medicines (negative outcomes associated with medication [NOM]) and situations in which the use of medicines caused or might cause the appearance of a NOM (drug-related problems). If necessary, lifestyle interventions to improve adherence and/or use of drug therapy were implemented.
- Completed a new assessment form. Completion of an intervention should have generated a change in the patient’s assessment. Depending on whether a NOM still existed, the therapeutic plan was completed.

ARV drugs were dispensed monthly and patient drug adherence was assessed using a simplified medication adherence questionnaire (SMAQ), an indirect method that has been validated in patients with HIV [22]. The SMAQ consists of six questions that assess different kinds of patient drug adherence:

- Do you ever forget to take your medicine?
- Are you careless at times about taking your medicine?
- Sometimes if you feel worse, do you stop taking your medicines?
- Thinking about the last week. How often have you not taken your medicine?
- Did you not take any of your medicine over the past weekend?
- Over the past 3 months, how many days have you not taken any medicine at all?

Patients were classified as non-adherent if they replied to any of the questions with a non-adherence answer. For quantification of omissions, patients were classified as non-adherent if they lost more than two doses during the previous week or had not taken medication for more than 2 complete days during the previous 3 months. Patients who were classified as non-adherent were excluded from the study.

To minimize potential biases, all blood samples were handled in the same manner and analysis was performed by the same certified laboratory using validated techniques. The ARV drugs, with their respective quality assurance certifications, were provided by Humax Pharmaceutical S.A.

### Statistical analysis

Statistical analyses were performed using STATA/IC 13.1 (Stata Corp LP, College Station, Texas, USA). An exploratory data analysis was used to calculate the central tendency measures, dispersion, frequency tables, 95 % confidence intervals, and normality tests. Data were reported as percentages or medians [quartile 1 (Q1)–quartile (Q3)]. Wilcoxon matched-pairs signed-rank test was used to compare proportions or medians. Comparisons were analyzed using two-tailed tests, and *p* < 0.05 was considered statistically significant.

The primary analysis was the proportion of patients who viral load <40 copies/mL at 12 months after baseline to assess effectiveness. Both intention to treat (ITT) analyses (consider all the subjects included at the beginning of the study) and on treatment (OT) analyses (consider only the subjects who fulfill completed the 12 months of followed) were used to analyze the results.

### Ethical approval

The study was conducted according to the ethical principles regarding human experimentation established in Colombia by both Resolutions 8430 of 1993 and 2378 of 2008, as the “*Declaration of Helsinki*” and its amendments, and the Guide to Good Clinical Practice (ICH E6). Informed consent was obtained from each participant before inclusion in the study. The study was approved by the medical ethical committee from the institution participating (Comité de Ética para Investigación en Humanos de la Corporación de Lucha Contra el SIDA–IRB 00005732at U.S. Department of Health and Human Services).

## Results

**Fig. 1:**
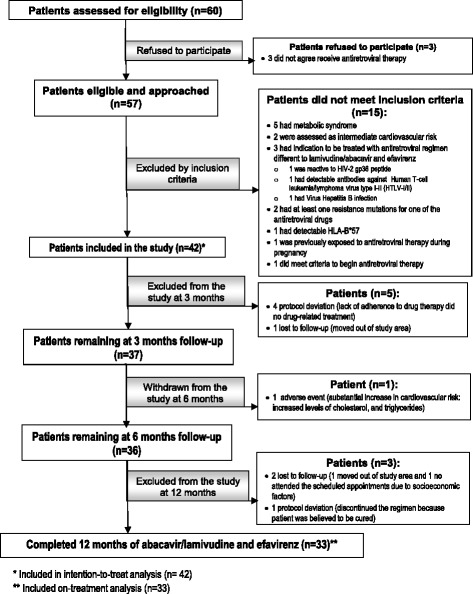
Flow chart of patients invited to participate in the generic drug study, Cali-Colombia, 2011–2012

A flowchart of the patients involved in the Cali-Colombia generic drug study is shown in Fig. 1. A group of 60 patients were invited to participate in the study; 42 were enrolled and 33 completed the 12 months of follow-up. Nine of the 42 patients presented reasons for excluding from the study (Fig. 1). The demographic characteristics of the 42 patients enrolled and 33 that completed the study are shown in Table 1. In patients who completed the study, the median age (Q1–Q3) was 37.0 (26.7–42.7) years; 21 (63.6 %) were male, 24 (72.7 %) were employed, and 27 (81.8 %) were identified as stage A (asymptomatic, acute HIV, or persistent generalized lymphadenopathy), according to the Centers for Disease Control and Prevention [23].

**Table 1:** Demographic characteristics of patients (enrolled *n* = 42, or completed the study *n* = 33), Cali-Colombia, 2011–2012

Patient characteristics		*n* = 42	*n* = 33
Age, Median (Q1-Q3)	Total	34.0 (24–43)	37.0 (26.7–42.7)
	Men	30.0 (24.0–41.0)	37.0 (26.9–42.4)
	Women	34.5 (26.0–44.0)	35.6 (24.6–44.9)
Gender, *n* (%)	Male	28 (66.7)	21 (63.6)
	Female	14 (33.3)	12 (36.4)
Ethnicity, *n* (%)	Black	7 (16.7)	6 (18.2)
	Nonblack	35 (83.3)	27 (81.8)
Marital status, *n* (%)	Married	13 (31.0)	12 (36.4)
	Single	29(69.0)	21 (63.6)
Education, *n* (%)	Elementary	10 (23.8)	8 (24.2)
	High school	17 (40.5)	13 (39.4)
	Technical education	6 (14.3)	5 (15.2)
	University	9 (21.4)	7 (21.2)
Occupation, *n* (%)	Home	5 (11.9)	4 (12.1)
	Student	6 (14.3)	3 (9.1)
	Unemployed	3 (7.1)	2 (6.1)
	Employed	28 (66.6)	24 (72.7)
Centers for Disease Control and Prevention clinical stages	A	35 (83.3)	27 (81.8)
	B	6 (14.3)	5 (15.2)
	C	1 (2.4)	1 (3.0)

*Q* quartile

### Effectiveness of generic version of abacavir/lamivudine and efavirenz

Regarding effectiveness at 12 months, 31 of 42 patients—73.8 % in intention-to-treat analysis; and 31 of 33 patients—93.9 % on-treatment analysis—achieved a viral load of HIV1 RNA <40 copies/mL (Table 2). Between baseline and 12 months, there were significantly increases of 172 in the median (Q1–Q3) of CD4 T lymphocyte count [373 (326–483) cells/mm^3^ vs. 545 (439–634) cells/mm^3^, (*p* <0.001)], and of 12.9 % in median (Q1–Q3) of %CD4 [26.7 (20.0–32.7) vs. 39.6 (34–47), (*p* <0.001)]. Similarly, there was a statistically significant increase in the CD4 T-lymphocyte count/CD8 T-lymphocyte count ratio above 0.5.

**Table 2:** Clinical variables related to drug effectiveness assessment, (enrolled *n* = 42, or completed the study *n* = 33), Cali-Colombia, 2011–2012

Clinical variable	Baseline		3 months	6 months	12 months
Viral load copies/mL	*n* = 42	*n* = 33	*n* = 33	*n* = 33	*n* = 33
Median (Q1–Q3)	18,013 (8236–44,936)	16,976 (7181–34,505)	0 (0-0)	0 (0-0)	0 (0-0)
≤40 copies, *n* (%)^a^	0 (0.0)	0 (0.0)	28 (84.8)	31 (93.9)	31 (93.9)
41-400 copies, *n* (%)	0 (0.0)	0 (0.0)	5 (15.2)	2 (6.1)	2 (6.1)
401-1.000 copies, *n* (%)	1 (2.4)	1 (3.0)	0 (0.0)	0 (0.0)	0 (0.0)
1.001-100.000 copies, *n* (%)	41 (97.6)	32 (97.0)	0 (0.0)	0 (0.0)	0 (0.0)
CD4 lymphocyte count (cell/mm^3^)					
≥ 200 n (%)	39 (92.9)	31 (95.9)	33 (100.0)	33 (100.0)	33 (100.0)
Median (Q1–Q3)^a^	373 (326–483)	375 (333–491)	532 (426–587)	509 (390–558)	545 (439–634)
%CD4 lymphocyte					
Median (Q1–Q3)^a^	26.7 (20.0–32.7)	26.7 (23.2–32.4)	34.6 (27.0–37.7)	37.6 (30.6–40.3)	39.6 (34–47)

*Q* quartile

^a^ *p*-value <0.0011, for differences between baseline and 3, 6, and 12 months

### Safety of generic version of abacavir/lamivudine and efavirenz

During 12 months of follow-up no meaningful or significant changes were seen in renal, hepatic, and hematologic function in the patients included and that completed the study (Table 3). In addition, of the 42 patients included in the study, 9 (21.4 %) presented reasons for excluded from the study. While no CV events were observed, one cART-related adverse event leading to withdraw in one patient (2.4 %) was due to increased CV risk (increased levels of cholesterol, triglycerides, and hs-CRP). Exclusion of the other eight patients from the study was owing to: dropouts not associated with ARV therapy, four patients (9.5 %) and non-adherence to ARV drugs not associated with safety problems, four patients (9.5 %), identified during the initial 3 months of follow-up (Fig. 1). Thus, among the 42 patients included in the study, and the 37 adverse events considered related to the study, cART was the cause of withdrawal in only one case. The most frequently reported adverse events were dizziness (7 patients), skin rash (6 patients), and drowsiness (5 patients) (Table 4). Abacavir was not associated with immunologically mediated hypersensitivity reaction, thus patients did not presented with this kind of adverse effect.

**Table 3:** Clinical variables related to drug safety assessment of patients (enrolled *n* = 42, or completed the study *n* = 33), Cali-Colombia, 2011–2012

	Baseline		6 months	12 months	*p* value^a^
Clinical variable	*n* = 42	*n* = 33	*n* = 33	*n* = 33	
Transaminase (ALT) U/L					
Median (Q1–Q3)	23 (18–32)	23.0 (17–32)	26.0 (18–41)	30.0 (20–44)	0.0452
Transaminase (AST) U/L					
Median (Q1–Q3)	22 (19–34)	22.0 (20–36)	25.0 (20–29)	26.0 (20–32)	0.8792
Serum creatinine (mg/dL)					
Median (Q1–Q3)	1.04 (0.97–1.17)	1.05 (0.98–1.17)	0.97 (0.90–1.06)	0.98 (0.90–1.09)	0.0895
Hemoglobin (g/dL)					
Median (Q1–Q3)	14.9 (13.1–15.8)	14.4 (12.9–15.5)	14.7 (13.5–15.2)	14.6 (13.4–15.8)	0.1983
Fasting total cholesterol (mg/dL)					
Median (Q1–Q3)	161.0 (140.0–177.0)	164.0 (140–179)	212.0 (191–231)	226.0 (200–246)	<0.001
≤ 200, *n* (%)	39 (92.9)	30 (90.9)	13 (39.4)	9 (27.3)	<0.001
200–240, *n* (%)	2 (4.8)	2 (6.1)	15 (45.4)	14 (42.4)	
> 240, *n* (%)	1 (2.4)	1 (3.0)	5 (15.2)	10 (30.3)	
Low-density lipoprotein cholesterol –[LDL-C]– (mg/dL)					
Median (Q1–Q3)	99.8 (87.4–114.9)	97.3 (87.4–114.9)	132.2 (116.1–152.3)	136.6 (115.3–155.1)	<0.001
≤ 130, *n* (%)	37 (88.1)	29 (87.9)	16 (48.5)	13 (39.4)	<0.001
130–160, *n* (%)	5 (11.9)	4 (12.1)	11 (33.3)	13 (39.4)	
> 160, *n* (%)	0 (0.0)	0 (0.0)	6 (8.2)	7 (21.2)	
High-density lipoprotein cholesterol –[HDL-C]– (mg/dL)					
Median (Q1–Q3)	38.4 (32.7–44.5)	38.4 (33.4–46.1)	47.0 (38.6–56.6)	51.7 (42.8–60.3)	<0.001
< 40for men or <50 for women, *n* (%)	27 (64.3)	18 (54.6)	9 (27.3)	6 (18.1)	0.0005
≥ 40 for men or ≥50 for women, *n* (%)	15 (35.7)	15 (45.4)	24 (72.3)	27 (81.8)	
Total cholesterol/ HDL-cholesterol					
Median (Q1–Q3)	4.2 (3.7–5)	4.0 (3.7–4.9)	4.4 (3.8–5.0)	4.3 (3.5–5.1)	0.2348
< 5, *n* (%)	32 (76.2)	25 (75.8)	25 (75.8)	25 (75.8)	
≥ 5, *n* (%)	10 (23.8)	8 (24.2)	8 (24.2)	8 (24.2)	
Triglycerides (mg/dL)					
Median (Q1–Q3)	91 (76–126)	95 (76–126)	139 (99–179)	146 (105–208)	<0.001
< 200, *n* (%)	40 (95.2)	32 (97.0)	27 (81.8)	24 (72.7)	0.0047
≥ 200, *n* (%)	2 (4.8)	1 (3.0)	6 (18.2)	9 (27.3)	
High-sensitivity C-reactive protein (mg/L)					
Median (Q1–Q3)	1.6 (1.1–3.4)	1.8 (1.2–3.4)	2.6 (1.1–4.5)	2.3 (1–5.1)	0.0635
Glycemia (mg/L)					
Median (Q1–Q3)	88 (82–94)	88 (81–95)	89 (86–95)	91 (85–97)	0.0106
Waist circumference (cm)					
Median (interquartile range)	85 (77–91)	85 (77–91)	84 (78–89)	84 (76–90)	0.6888
Cardiovascular risk according to Framingham score					
Median (Q1–Q3)	−1 (−1-1)	−1 (−1-1)	1 (−1–2)	1 (−1–3)	0.0002
< 1, *n* (%)	26 (63.4)	20 (60.6)	14 (42.4)	16 (48.5)	0.0002
1, *n* (%)	9 (21.9)	8 (24.2)	9 (27 .3)	5 (15.2)	
2, *n* (%)	4 (9.8)	3 (9.1)	5 (15.2)	3 (9.1)	
3, *n* (%)	1 (2.4)	1(3.0)	1 (3.0)	3 (9.1)	
4, *n* (%)	0 (0.0)	0 (0.0)	0 (0.0)	1 (3.0)	
5, *n* (%)	0 (0.0)	0 (0.0)	1 (3.0)	2 (6.1)	
6, *n* (%)	1 (2.4)	1 (3.0)	3 (9.1)	1 (3.0)	
8, *n* (%)	0 (0.0)	0 (0.0)	0 (0.0)	1 (3.0)	
10, *n* (%)	0 (0.0)	0 (0.0)	0 (0.0)	1 (3.0)	

*Q* quartile

^a^For differences between baseline and 12 months

**Table 4:** Adverse events observed in patients treated with generic abacavir/lamivudine and efavirenz in comparison to adverse events reported in published clinical trials evaluating the same branded drugs

Adverse event in studies with regimen abacavir/lamivudine/efavirenz	Current study, *N* = 42	CLASS Study [20] *N* = 97 (96 weeks)	CNA30024 Study [21], *N* = 324	ESS30009 Study [22], *N* = 169
Skin rash, *n* (%)	6 (14.3)	7 (7.2)	21 (6.5)	16 (9.6)
Dizziness, *n* (%)	7 (16.7)	No reported	18 (5.6)	9 (5.4)
Drowsiness, *n* (%)	5 (11.9)	No reported	No reported	No reported
Depressive disorder, *n* (%)	3 (7.1)	No reported	20 (6.2)	8 (4.8)
Sleep disorder (insomnia), *n* (%)	3 (7.1)	8 (8.2)	20 (6.2)	10 (5.9)
Dreams, *n* (%)	2 (4.8)	6 (6.2)	15 (4.6)	No reported
Anxiety, *n* (%)	1 (2.4)	No reported	No reported	4 (2.4)
Tingling/Numbness (Sensation of heat)	3 (7. 1)	No reported	No reported	No reported
Dyspepsia or abdominal pain, *n* (%)	3 (7.1)	No reported	12 (3.7)	No reported
Nausea, *n* (%)	1 (2.4)	4 (4.1)	22 (6.8)	No reported
Anorexia	1 (2.4)	No reported	No reported	No reported
Fatigue/weakness, *n* (%)	1 (2.4)	1 (2.4)	No reported	7 (4.2)
Increased cardiovascular risk - *leading to study drug discontinuation*	1 (2.4)	No reported	No reported	No reported
Vomiting, *n* (%)	0 (0.0)	No reported	7 (2.2)	No reported
Suspected hypersensitivity to abacavir or other drug, *n* (%)	0 (0.0)	8 (8.2)	28 (8.6)	17 (10.1)
Headache, *n* (%)	0 (0.0)	No reported	19 (5.9)	10 (5.9)
Ear, nose, and throat (ENT) Infections, *n* (%)	0 (0.0)	No reported	29 (9.0)	11 (6.6)
Upper respiratory tract infection, *n* (%)	0 (0.0)	No reported	13 (4.0)	9 (5.4)
Diarrhea, *n* (%)	0 (0.0)	1 (1.0)	22 (6.8)	6 (3.6)
Viral respiratory infection, *n* (%)	0 (0.0)	No reported	21 (6.5)	No reported
Musculoskeletal pain, *n* (%)	0 (0.0)	No reported	21 (6.5)	No reported
Hypertriglyceridemia, *n* (% among 33 finished the study)	8 (24.2)	9 (9.3)	18 (5.6)	No reported
Increased Creatinine phosphokinase, *n* (%)	No assessed	No reported	No reported	19 (11.4)
Increased liver function test results, *n* (%)	0 (0.0)	No reported	13 (4.0)	14 (8.4 %)
Anemia, *n* (%)	0 (0.0)	No reported	7 (2.2)	No reported

### Lipids and cardiovascular risk

Patients included in the study showed significant increases in TC, low-density lipoprotein cholesterol (LDL-C), HDL-C, and triglycerides. There was a significant increase of 2 %, in the median of the percentage of CV risk score, according to the Framingham equation (from −1 % to 1 %); however, at 48 weeks the CV risk score for 33 patients continued to be valued as lower risk, and only 1 patient was assessed as 10 % of CV risk (Table 3).

## Discussion

The current study describe the effectiveness and safety ABC/3TC and EFV generic version for the treatment of HIV/AIDS under the real-world clinical setting of a Cali, Colombia medical practice. The findings showed that in treatment-naïve HIV-infected patients 18 years or older with indication to receive cART, without primary or transmitted resistances, attending a program that provides comprehensive consultation and continuing care, generic ABC/3TC and EFV was effective and safe as expected based on the proprietary drug scheme. The findings are similar to those reported in a study designed to assess the safety and effectiveness data from the generic drug scheme lamivudine/zidovudine and efavirenz in HIV/AIDS treatment naïve patients [14]. So, in another study assessed the long-term safety, effectiveness, and quality of the generic fixed-dose combination of nevirapine, stavudine and lamivudine [24]. These types of studies will continue to assist generic drug companies in bringing to market effective and safe ARV drugs, as information becomes available regarding the effectiveness and safety of approved drugs.

### Effectiveness

Ideally, it would be to compare the change form baseline CD4 cells/mm^3^ and HIV RNA copies/mL with another arm or a less with results obtained in a group with similar conditions [14]. Thus, we conducted a review in PubMed/Medline to identify articles with clinical results in patients with HIV/AIDS in treatment with ABC/3TC and EFV original drugs. Search terms were: abacavir/lamivudine and efavirenz. Thus, we identified the main articles with results associate to use the scheme ABC/3TC and efavirenz, including the published generate from AIDS Clinical Trials Group A5202 [25–27]. However, both significant difference in baseline CD4 cells/mm^3^ as in values of HIV RNA copies/mL in our study limited to use these results as a possible indirect comparator arm [14]. For instance in the Study A5202 [26], in the abacavir/lamivudine and efavirenz arm (*n* = 465), both the baseline median CD4 count (Q1–Q3) [225 (103–324) cells/mm^3^] and the baseline median HIV-1 RNA level (Q1–Q3), [4.7 (4.3–5.0) log10 copies/mL] different notorious from our baseline values median CD4 count (Q1–Q3) [373 (326–483) cells/mm^3^] and median HIV-1 RNA level (Q1–Q3) [(4.3 (3.9–4.7) log10 copies/mL].

Currently, it is expected that initial cART has a virologic response rate higher than 90 % at 12 months [28]. In this study 74 % of patients (intention-to-treat analysis) in treatment with ABC/3TC and EFV obtained undetectable viral load, a value similar to those reported in other studies (75 % [29], 72 % [30], and 71 % [31]) of patients in treatment with this ARV scheme; but it was higher than 64 % [32], and 57 % [33] reported in other studies (Table 5). There was a significant increase (172 cells/mm^3^) in the median for CD4 T lymphocyte count (Tables 2 and 5), a value that was closer to those reported for several studies in which the increases in the median for CD4 T lymphocyte count ranged between 130 and 209 cells/mm^3^ (Table 5).

**Table 5:** Effectiveness of abacavir/lamivudine and efavirenz (viral load non-detectable and increase in median of CD4) in HIV naive patients 18 years or older, compared with six other HIV/AIDS drug treatment studies

Type of study	Patients (n)	% of patients with viral load <50 copies/mL		Median CD4+ cell count increases [cells/mm^3^]	Commentary
		Intention-to-treat analysis	On-treatment analysis		
Nonrandomized, open-label, phase IV study (*Current Study,* viral load <40 copies/mL))	42	73.8	93.9	172	HLA-B*57 allele negative. 91.2 % patients with viral load <40 copies/mL, in intention-to-treat analysis; Failure
Open-label, multicenter, randomized trial of up to 3 consecutive treatment regimens over 96 weeks in 291 subjects received abacavir/lamivudine and efavirenz, ritonavir-boosted amprenavir, or stavudine (*CLASS Study*) [29]	291	75 (estimated values from figure 3C at 48 week)	91 (64 of 70 patients received abacavir/lamivudine and efavirenz)	194	HLA-B*5701 genotype test was not screening. Results included patients with viral load <100.000 or > 100.000 copies/mL
Multicenter, randomized, double-blind no inferiority clinical trial of abacavir with zidovudine plus lamivudine and efavirenz (*CNA30024 Study*) [30]	324	71.7 (142 of 198 patients with viral load <100.000 copies/mL)	89.3 % (226 of 253 patients with viral load <100.000 or > 100.000 copies/mL)	209	HLA-B*5701 genotype test was not screening.
Randomized, open-label, multicenter study of abacavir/lamivudine administered with tenofovir or efavirenz (*ESS30009 Study*) [31]	169	71.0	94.5	130	HLA-B*5701 genotype test was not screening. The majority of subjects in the tenofovir arm switched regimens or withdrew before week 16
Multicenter, randomized, open-label study of abacavir/lamivudine or tenofovir/emtricitabine administered with efavirenz (*ASSERT Study*) [32]	192	64.2 (61 of 95 patients with viral load <100.000 copies/mL)	N/D	150	HLA-B*5701 genotype negative. Viral load suppression as secondary efficacy endpoints
Multicenter and randomized study of efavirenz with abacavir/lamivudine or lopinavir/r with abacavir/lamivudine (*Lake Study*) [33]	63	56.7	87.0	193	HLA-B*5701 genotype test was not screening. Results included patients with viral load <100.000 or > 100.000 copies/mL
Multicenter retrospective study of tenofovir-emtricitabine or abacavir-lamivudine, administered with efavirenz for 260 weeks (*TOKEN Study*) [40]	75	N/D	85	135	HLA-B*5701 genotype negative. Estimated values at 48 week from Fig. 1a (viral load <40 copies/mL) and Fig. 1b (CD4 count)

### Safety

The most frequently identified adverse events related to ARV drugs in the 42 patients were dizziness (16.7 %), skin rash (14.3 %), and drowsiness (11.9 %) (Table 4). ABC was not associated with immunologically mediated hypersensitivity reaction. During the 12 months of follow-up, there were no significantly changes in renal, hepatic, or hematologic function among the 33 patients who completed the study (Table 3). The safety profile of the generic version of ABC/3TC and EFV (Tables 3 and 4) was similar to that reported for the proprietary ARV drugs (during 12 months of follow-up), especially with the increase of TC, LDL-C, HDL-C, and the CV risk. However, the frequency of some types of adverse events such as skin rash, dizziness, and hypertriglyceridemia was higher compared with other studies (Tables 3 and 4). This difference could be explained by closer and continuing follow-up of patients by the pharmacist and the smaller number of patients in the study.

It is important to note that of the 42 patients included in the study, only one experienced a cART-related adverse event leading to withdrawn, specifically due to increased CV risk. The other eight causes for excluding were for reasons not associated with ARV therapy (four patients) and non-adherence to ARV drugs (four patients) did no drug-related treatment. A total of 37 adverse events considered related to the study were reported for the 42 patients but cART only was the cause of withdrawal for just one patient, 2.4 % (1 of 42). Compared with other studies that used ABC/3TC and EFV proprietary scheme, this value is lower than 4.9 % (16 of 324 patients) [30], 10.7 % (18 of 169 patients) [31], 13.0 % (25 of 192 patients) [32], and 22.2 % (14 of 63 patients; 6 discontinued due to a hypersensitivity reaction/rash) [33]. These differences could be caused by drug hypersensitivity, including ABC hypersensitivity reaction, mainly in studies where HLA-B*5701 (or HLA-B*57) genotype test was not screening at baseline [30, 31, 33]. In our study neither patient presented ABC hypersensitivity reaction. This was expected because the patients received genetic screened for the HLA-B*57 allele, an intervention that would decrease the incidence of this adverse event. Patients who test negative with HLA-B*57 screening comprise 90–95 % of HIV-1-infected patients and require no further HLA-B*5701 confirmation by molecular HLA method [34]. HLA-B*57 screening is a low-cost alternative to high-resolution typing of patients, and lends itself to point-of-care diagnostics and rapid assessment of low-risk patients who can begin immediate therapy with ABC [35].

Both ABC/3TC [32, 36] and EFV [37, 38] may lead to modest rises in TC, LDL-C, HDL-C, and triglycerides. Our study showed statistically significant increases in medians of 65.0 mg/dL in TC, of 36.8 mg/dL in LDL-C, of 13.3 mg/dL in HDL-C, and of 55.0 mg/dL in triglycerides (Table 3). Except for triglycerides, these increases were similar to those reported in other studies. For instance, in the Lake study there were significant increases of 48 mg/dL in TC, and of 10 mg/dL in HDL-C [33]. In another study with 75 patients, over 96 weeks there was an increase of 42.0 mg/dL in TC, of 21.6 mg/dL in LDL-C, of 25 mg/dL in triglycerides, and of 18 mg/dL in HDL-C [39]. However, these rises in cholesterol and triglycerides are not of sufficient magnitude to influence CV risk. For example, in our study the median of percentage of CV risk based on the Framingham Equation [20] at baseline versus 48 weeks of follow-up showed an increase of only 2 % (from −1 % to 1 %); thus, at 48 weeks, in the 33 patients, CV risk continued to be low (Table 3). Similarly, in the TOKEN study (*n* = 178 patients at baseline) [40] there was an increase of 0.9 % in CV risk (baseline vs. 48 weeks, from 2.6 to 3.5 %).

The increase in the CV risk may be proportional to the increase in TC levels, LDL-C, and triglycerides. However, ABC has been associated only to slight increase of the CV risk, and more important the data related to ABC, as a cause of myocardial infarction, remain inconclusive and controversial. In this way, some studies have concluded that an increased risk of myocardial infarction exists in patients exposed to ABC in the preceding 6 months [41] and it has been associated with increase of the CV risk [42]; however, myocardial infarction associated to ABC has not been seen in randomized clinical trials or meta-analysis [43, 44].

This study provides important elements for evaluation regarding the effectiveness and safety of generic ARVs produced in Colombia. Phase IV clinical trials could be an important option to assess and support the quality of generic drugs used in standardized clinical practice [14]. Our findings do not necessarily indicate therapeutic equivalence between ABC/3TC and EFV generic and proprietary ARV, but they do support the quality and use of this generic version. In developing countries, including Colombia, to guarantee the long-term sustainability of access to ART it is necessary to develop and support the generic market [7, 8].

Efforts are need to reduce drug costs through increased use of generic drugs, price negotiation, and public health directives such as compulsory licensing [45]. Colombia is considered as a country on resource and capacity limited settings, and it is expected that the use of generics may improve the coverage to ARV drugs [4]. Although bioequivalence of ART regimen composed by ABC/3TC and EFV generics have been determined according to Colombia regulations [13], the results of this study may contribute both to generate trust in the quality of the generic drugs and to improve the coverage to ARV drugs, due to increase of use of generic ART manufactured in Colombia. In this way, it is important to denote that the 9.7 million people receiving ART in low- and middle-income countries, for instance Colombia, represents only 34 % (32–37 %) of the 28.6 (26.5–30.9) million people eligible in 2013 [1].

### Study limitations

Because our study had several limitations, the results and conclusions should be interpreted with caution. First, this study was based on a small sample of HIV/AIDS treatment naïve patients who were followed for only 12 months. Thus, the small sample size could be insufficient to obtain strong conclusions. However, the current study may prove clinical information about effectiveness and safety of ABC/3TC and EFV generics, which has been considered as scarce [11]. Second, it was a nonrandomized, open-label, study; therefore it lacked a control group and causal effect could not be established. It is important to note that we did not find studies of patients in treatment with ABC/3TC and EFV original drugs and with similar viral load and lymphocytes CD4 count at baseline. In our study patients start cART with lower baseline viral load and upper lymphocytes CD4 count, compared with other studies. In addition, we have to use the results from studies in populations and endpoints different, which may generated some limitations. However, these studies proved an overall of clinic results obtained with scheme ABC/3TC and EFV original drugs. Finally, our study could be confounded by pharmaceutical care performed by a pharmacist trained in this practice and using the DADER method, oriented service mainly to monitoring drug outcomes, and counseling regarding adherence and appropriate use of ARV drugs by patients [17, 21], conditions that are critical in the first 3 months and may have improved clinical outcomes.

## Conclusions

In a small group of HIV-infected treatment naïve patients followed for 12 months, this open-label, nonrandomized study showed that, as expected, the clinical outcomes of generic drug version of ABC/3TC and EFV were compatible with the safety and effectiveness profile of proprietary ARV drugs.

## Abbreviations

3TC: Lamivudine
ABC: Abacavir
ART: Antiretroviral therapy
ARV: Antiretroviral
cART: Combination antiretroviral therapy
CV: Cardiovascular
CVD: Cardiovascular disease
EFV: Efavirenz
HDL-C: High-density lipoprotein cholesterol
HIV: Human immunodeficiency virus
HLA: Histocompatibility complex allele
hs-CRP: high-sensitivity C-reactive protein
LDL-C: Low-density lipoprotein cholesterol
NOM: Negative outcomes associated with medication
Q1–Q3: Quartile 1 – quartile 3
RNA: Ribonucleic acid
TC: Total cholesterol
WHO: World Health Organization

## References

[1] UNAIDS (2013). Global report: UNAIDS report on the global AIDS epidemic 2013.

[2] Cohen MS, Chen YQ, McCauley M (2011). Prevention of HIV-1 infection with early antiretroviral therapy. N Engl J Med.

[3] Palella FJ, Delaney KM, Moorman AC, Loveless MO, Fuhrer J, Satten GA (1998). Declining morbidity and mortality among patients with advanced human immunodeficiency virus infection. HIV outpatient study investigators. N Engl J Med.

[4] Ministerio de Salud y Protección Social de Colombia. Guía de práctica clínica basada en la evidencia científica para la atención de la infección por VIH/Sida en adolescentes (con 13 años de edad o más) y adultos; 2014. http://gpc.minsalud.gov.co/gpc_sites/Repositorio/Otros_conv/GPC_VIH_adolescentes/gpc_vih_adolescentes_completa.aspx. Accessed 19 Apr 2016.

[5] World Health Organization (2013). Consolidated guidelines on the use of antiretroviral drugs for treating and preventing HIV infection.

[6] Waning B, Kaplan W, King AC, Lawrence DA, Leufkens HG, Fox MP (2009). Global strategies to reduce the price of antiretroviral medicines: evidence from transactional databases. Bull World Health Organ.

[7] Danzon PM, Mulcahy AW, Towse AK (2015). Pharmaceutical pricing in emerging markets: effects of income, competition, and procurement. Health Econ.

[8] Meiners C, Sagaon-Teyssier L, Hasenclever L, Moatti JP (2011). Modeling HIV/AIDS drug price determinants in Brazil: Is generic competition a myth?. PLoS One.

[9] Chaves GC, Hasenclever L, Osorio-de-Castro CG, Oliveira MA (2015). Strategies for price reduction of HIV medicines under a monopoly situation in Brazil. Rev Saude Publica.

[10] Wang T, Hoag SW, Eng ML, Polli J, Pandit NS (2015). Quality of antiretroviral and opportunistic infection medications dispensed from developing countries and internet pharmacies. J Clin Pharm Ther.

[11] Yoosakul E, Vatanarongkup J, Manamuti C, Chuasuwan B, Techatanawat I, Karacho B (2016). Bioequivalence study of abacavir/lamivudine (600/300-mg) tablets in healthy Thai volunteers under fasting conditions. Asian J. Pharm. Sci.

[12] Al Ameri MN, Nayuni N, Anil Kumar KG, Perrett D, Tucker A, Johnston A (2011). The differences between the branded and generic medicines using solid dosage forms: in-vitro dissolution testing. Results Pharma Sci.

[13] Ministerio de Salud de Colombia (2001). Resolución 1400.

[14] Gutiérrez FJ, Amariles P, Galindo J, Mueses H, Agudelo JA, Hincapié JA (2013). Efectividad y seguridad del esquema genérico Lamivudina/Zidovudina/Efavirenz en pacientes VIH (+). Estudio fase IV y comparación con el mismo esquema de medicamentos innovadores. Revista Vitae.

[15] Stringer JS, Mwango AJ, Giganti MJ, Mulenga L, Levy JW, Stringer EM (2012). Effectiveness of generic and proprietary first-line anti-retroviral regimens in a primary health care setting in Lusaka, Zambia: a cohort study. Int J Epidemiol.

[16] Amariles P, Faus MJ, Sabater D, Machuca M, Martinez-Martinez F (2006). Seguimiento Farmacoterapéutico y parámetros de efectividad y seguridad de la farmacoterapia. El farmacéutico.

[17] Amariles P (2011). Seguimiento Farmacoterapéutico en pacientes VIH/SIDA. Aula de Farmacia.

[18] Fleiss JL (1981). Statistical methods for rates and proportions.

[19] Thompson MA, Aberg JA, Cahn P, Montaner JS, Rizzardini G, Telenti A, International AIDS Society-USA (2010). Antiretroviral treatment of adult HIV infection: 2010 recommendations of the International AIDS Society-USA panel. JAMA.

[20] Expert Panel on Detection, Evaluation, and Treatment of High Blood Cholesterol in Adults (2001). Executive summary of the third report of the national cholesterol education program (NCEP) expert panel on detection, evaluation, and treatment of high blood cholesterol in adults (adult treatment panel III). JAMA.

[21] Pharmaceutical Care Research Group, University of Granada (Spain) (2006). Pharmacotherapy follow-up: the dader method (third revision: 2005). Pharm Pract.

[22] Knobel H, Alonso J, Casado JL, Collazos J, Gonzalez J, Ruiz I (2002). Validation of a simplified medication adherence questionnaire in a large cohort of HIV-infected patients: the GEEMA Study. AIDS.

[23] CDC. 1993 Revised Classification System for HIV Infection and Expanded Surveillance Case Definition for AIDS Among Adolescents and Adults. MMWR Recomm Rep.1992 Dec; 41(RR-17):1–19. 1361652

[24] Laurent C, Kouanfack C, Koulla-Shiro S, Njoume M, Nkene YM, Ciaffi L (2007). Long-term safety, effectiveness and quality of a generic fixed-dose combination of nevirapine, stavudine and lamivudine. AIDS.

[25] Sax PE, Tierney C, Collier AC, Fischl MA, Mollan K, Peeples L, AIDS Clinical Trials Group Study A5202 Team (2009). Abacavir-lamivudine versus tenofovir-emtricitabine for initial HIV-1 therapy. N Engl J Med.

[26] Daar ES, Tierney C, Fischl MA, Sax PE, Mollan K, Budhathoki C (2011). Atazanavir plus ritonavir or efavirenz as part of a 3-drug regimen for initial treatment of HIV-1: a randomized trial. Ann Intern Med.

[27] Sax PE, Tierney C, Collier AC, Daar ES, Mollan K, Budhathoki C (2011). Abacavir/lamivudine versus tenofovir DF/emtricitabine as part of combination regimens for initial treatment of HIV: final results. J Infect Dis.

[28] Gatell Artigas JM (2010). HIV infection. Irreversible mistakes not to be repeated. Med Clin (Barc).

[29] Bartlett JA, Johnson J, Herrera G, Sosa N, Rodriguez A, Liao Q, Clinically Significant Long-Term Antiretroviral Sequential Sequencing Study (CLASS) Team. Long-term results of initial therapy with abacavir and Lamivudine combined with Efavirenz, Amprenavir/Ritonavir, or Stavudine. J Acquir Immune Defic Syndr. 2006;43(3):284–92. 10.1097/01.qai.0000243092.40490.26 16967040

[30] DeJesus E, Herrera G, Teofilo E, Gerstoft J, Buendia CB, Brand JD, CNA30024 Study Team, et al. Abacavir versus zidovudine combined with lamivudine and efavirenz, for the treatment of antiretroviral-naive HIV-infected adults. Clin Infect Dis. 2004;39(7):1038–46. 10.1086/424009 15472858

[31] Gallant JE, Rodriguez AE, Weinberg WG, Young B, Berger DS, Lim ML, ESS30009 Study (2005). Early virologic nonresponse to tenofovir, abacavir, and lamivudine in HIV-infected antiretroviral-naive subjects. J Infect Dis.

[32] Post FA, Moyle GJ, Stellbrink HJ, Domingo P, Podzamczer D, Fisher M, et al. Randomized comparison of renal effects, efficacy, and safety with once-daily abacavir/lamivudine versus tenofovir/emtricitabine, administered with efavirenz, in antiretroviral-naive, HIV-1-infected adults: 48-week results from the ASSERT study. J Acquir Immune Defic Syndr. 2010;55(1):49–57. 10.1097/QAI.0b013e3181dd911e 20431394

[33] Echeverría P, Negredo E, Carosi G, Gálvez J, Gómez JL, Ocampo A, et al. Similar antiviral efficacy and tolerability between efavirenz and lopinavir/ritonavir, administered with abacavir/lamivudine (Kivexa), in antiretroviral-naïve patients: a 48-week, multicentre, randomized study (Lake Study). Antiviral Res. 2010;85(2):403–8. 10.1016/j.antiviral.2009.11.008 19941906

[34] Rodriguez-Sainz C, Valor L, Hernandez DC, Gil J, Carbone J, Pascual-Bernaldez M (2013). Flow cytometry analysis with a new FITC-conjugated monoclonal antibody-3E12 for HLA-B*57:01 rapid screening in prevention of abacavir hypersensitivity in HIV-1-infected patients. HIV Clin Trials.

[35] Kostenko L, Kjer-Nielsen L, Nicholson I, Hudson F, Lucas A, Foley B (2011). Rapid screening for the detection of HLA-B57 and HLA-B58 in prevention of drug hypersensitivity. Tissue Antigens.

[36] Smith KY, Patel P, Fine D, Bellos N, Sloan L, Lackey P, HEAT Study Team (2009). Randomized, double-blind, placebo matched, multicenter trial of abacavir/lamivudine and tenofovir/emtricitabine with lopinavir/ritonavir for initial HIV treatment. AIDS.

[37] Kirchner JT (2012). A tolerability review of non-nucleoside reverse transcriptase inhibitors: focus on laboratory measures of clinical relevance. J Antivir Antiretrovir.

[38] Quercia R, Roberts J, Martin-Carpenter L, Zala C (2015). Comparative changes of lipid levels in treatment-naive, HIV-1-infected adults treated with dolutegravir vs. efavirenz, raltegravir, and ritonavir-boosted darunavir-based regimens over 48 weeks. Clin Drug Investig.

[39] Podzamczer D, Ferrer E, Sanchez P, Gatell JM, Crespo M, Fisac C, ABCDE (Abacavir vs. d4T (stavudine) plus efavirenz) Study Team (2007). Less lipoatrophy and better lipid profile with abacavir as compared to stavudine: 96-week results of a randomized study. J Acquir Immune DeficSyndr.

[40] Pammi M, Arumainayagam J, Kumari B, Ahmed-Jushuf I, Carlin EM, Chandramani S (2013). Safety and efficacy of tenofovir/emtricitabine or abacavir/lamivudine in combination with efavirenz in treatment naïve HIV patients: a 5 year retrospective observational cohort study (the TOKEN Study). Int J ClinPract.

[41] Sabin CA, Worm SW, Weber R, Reiss P, El-Sadr W, Dabis F, D:A:D Study Group (2008). Use of nucleoside reverse transcriptase inhibitors and risk of myocardial infarction in HIV-infected patients enrolled in the D:A:D study: a multi-cohort collaboration. Lancet.

[42] Strategies for Management of Anti-Retroviral Therapy/INSIGHT; DAD Study Groups (2008). Use of nucleoside reverse transcriptase inhibitors and risk of myocardial infarction in HIV-infected patients. AIDS.

[43] Bavinger C, Bendavid E, Niehaus K, Olshen RA, Olkin I, Sundaram V (2013). Risk of cardiovascular disease from antiretroviral therapy for HIV: a systematic review. PLoS One.

[44] Brothers CH, Hernandez JE, Cutrell AG, Curtis L, Ait-Khaled M, Bowlin SJ, et al. Risk of myocardial infarction and abacavir therapy: no increased risk across 52 Glaxo SmithKline sponsored clinical trials in adult subjects. J Acquir Immune Defic Syndr. 2009;51:20–8. 10.1097/QAI.0b013e31819ff0e6 19282778

[45] Luo J, Oliveira MA, Ramos MB, Maia A, Osorio-de-Castro CG (2014). Antiretroviral drug expenditure, pricing and judicial demand: an analysis of federal procurement data in Brazil from 2004–2011. BMC Public Health.

